# National cohort study of absolute risk and age-specific incidence of multiple adverse outcomes between adolescence and early middle age

**DOI:** 10.1186/s12889-015-2249-5

**Published:** 2015-09-19

**Authors:** Pearl L.H. Mok, Sussie Antonsen, Carsten Bøcker Pedersen, Louis Appleby, Jenny Shaw, Roger T. Webb

**Affiliations:** Centre for Mental Health and Safety, University of Manchester, Jean McFarlane Building, Oxford Road, Manchester, M13 9PL UK; Centre for Integrated Register-based Research, CIRRAU, Aarhus University, Aarhus, Denmark; National Centre for Register-Based Research, Aarhus University, Business and Social Sciences, Aarhus, Fuglesangs Alle 4, 8210 Aarhus V, Denmark

## Abstract

**Background:**

Psychiatric illness, substance misuse, suicidality, criminality and premature death represent major public health challenges that afflict a sizeable proportion of young people. However, studies of multiple adverse outcomes in the same cohort at risk are rare. In a national Danish cohort we estimated sex- and age-specific incidence rates and absolute risks of these outcomes between adolescence and early middle age.

**Methods:**

Using interlinked registers, persons born in Denmark 1966–1996 were followed from their 15^th^ until 40^th^ birthday or December 2011 (*N* = 2,070,904). We estimated sex- and age-specific incidence rates of nine adverse outcomes, in three main categories: Premature mortality (all-causes, suicide, accident); Psychiatric morbidity (any mental illness diagnosis, suicide attempt, alcohol or drug misuse disorder); Criminality (violent offending, receiving custodial sentence, driving under influence of alcohol or drugs). Cumulative incidences were also calculated using competing risk survival analyses.

**Results:**

For cohort members alive on their 15^th^ birthday, the absolute risks of dying by age 40 were 1.99 % for males [95 % confidence interval (CI) 1.95–2.03 %] and 0.85 % for females (95 % CI 0.83–0.88 %). The risks of substance misuse and criminality were also much higher for males, especially younger males, than for females. Specifically, the risk of a first conviction for a violent offence was highest amongst males aged below 20. Females, however, were more likely than males to have a hospital-treated psychiatric disorder. By age 40, 13.25 % of females (95 % CI 13.16–13.33 %) and 9.98 % of males (95 % CI 9.91–10.06 %) had been treated. Women aged below 25 were also more likely than men to first attempt suicide, but this pattern was reversed beyond this age. The greatest gender differentials in incidence rates were in criminality outcomes.

**Conclusions:**

This is the first comprehensive assessment of the incidence rates and absolute risks of these multiple adverse outcomes. Approximately 1 in 50 males and 1 in 120 females who are alive on their 15th birthday will die by age 40. By examining the same cohort at risk, we compared risks for multiple outcomes without differential inter-cohort biases. These epidemiological profiles will inform further research into the pathways leading to these adverse events and future preventive strategies.

## Background

Successfully negotiating the transition from adolescence to the commencement of middle age can be a challenging experience for some young people. While most individuals pass through their teenage years and their twenties and thirties relatively smoothly and successfully a sizeable proportion do not. Many adverse outcomes, such as psychiatric disorder, suicidal behaviour, alcohol and drug misuse, criminal offending, and premature mortality, share similar aetiological pathways. For example, adolescent emotional instability and conduct problems have been linked with premature death, especially so for suicide and, to a lesser extent, accidental death [1]. Some young people who engage in antisocial behaviours, such as heavy drinking, driving whilst intoxicated by alcohol or drugs, and interpersonal violence, have increased risks of both injury and psychological disorders in mid-adulthood [2]. Individuals with mental illness not only have elevated risks of suicide [3], but also accidental death [4] and death from natural causes [5]. In addition, those who have had contact with the criminal justice system, even without custodial sentences or guilty verdicts, have been reported to have elevated suicide risk [6]. Risks appear especially high amongst those charged with or convicted of violent offences [6–8], and those with a history of serious mental illness [9, 10], supporting the notion of a strong correlation between aggression against self and aggression against others, especially in younger people [11, 12].

Comparing the epidemiological profiles of these adverse outcomes will guide aetiological research into the causes of and pathways leading to these events. However, studies on multiple adverse events in the same cohort at risk are rare, making comparisons of these profiles difficult due to differential inter-cohort biases. National Scandinavian registers provide a virtually unique opportunity for undertaking this type of comparative multiple-outcome epidemiological study. In a national Danish cohort we estimated sex- and age-specific incidence rates and absolute risks of adverse events under three main outcome categories: 1. Premature mortality (all-causes, suicide, accident); 2. Psychiatric morbidity (any mental illness diagnosis, suicide attempt, alcohol or drug misuse disorder); 3. Criminality (violent offending, receiving a custodial sentence, convicted of driving under the influence of alcohol or drugs). We also estimated age-specific incidence rate differences comparing males versus females. From these analyses we wished to identify the greatest gender differentials across the array of adverse outcomes we examined.

## Methods

### Study population

Since 1968 the Civil Registration System (CRS) has registered all persons living in Denmark [13]. Among other variables, it captures personal identification numbers, gender, date and place of birth, and continuously updated information on vital status. The unique personal identification number is used in all national registers enabling accurate inter-register linkage. Our study population included all persons born in Denmark between January 1st 1966 and December 31st 1996 who were alive and residing in Denmark on their 15th birthday (*N* = 2,070,904).

## Ethics and consent statements

Approval to conduct this study was granted formally by the Danish Data Protection Agency (file number 2013–41–2265), and data access was agreed by the State Serum Institute and Statistics Denmark (FSE ID 820).

Since this project is based exclusively on register data it does not need approval from the Danish National Committee on Health Research Ethics. According to the Danish Act on Processing of Personal Data, section 10, this also means that the study is not required to obtain informed consent from persons in the study population.

### Measurement of adverse outcomes

Cohort members were linked via their personal identifier to various national registers to obtain information on the nine adverse outcomes of interest, as follows:

#### Premature mortality

The study cohort was linked with the Register of Causes of Death [14] to identify all-cause mortality [all International Classification of Diseases (ICD) death codes], suicides (ICD-8: E950–E959; ICD-10: X60-X84), and accidental deaths (ICD-8: E800-E929, E940-E942; ICD-10: V01-X59). This Register contains information for all residents who died in Denmark in 1970 or later. Danish legislation states that any sudden and unexpected death should be reported to the police and that in these circumstances the death certificate may only be issued after a medico-legal examination.

#### Psychiatric morbidity

Persons within the study cohort were linked via their personal identifier to the Psychiatric Central Research Register [15] and the National Patient Register [16] to obtain information on suicide attempts and history of mental illness. The Psychiatric Central Research Register was computerised in 1969 and contains data on all admissions to psychiatric inpatient facilities. The National Patient Register was established in 1977 and contains data on all admissions to public general hospitals. In both registers, information on outpatient visits was included from 1995 onwards. From 1969–1993, the diagnostic system used was the Danish modification of the International Classification of Diseases, 8th revision (ICD-8) [17], and from 1994 the diagnostic system used was the International Classification of Diseases, 10th revision, Diagnostic Criteria for Research (ICD-10-DCR) [18]. Onset of any psychiatric disorder (ICD8: 290–315; ICD10: F00-F99) and mental and behavioural disorders due to psychoactive substance abuse (ICD8: 291.×9, 294.39, 303.×9, 303.20, 303.28, 303.90, 304.×9; ICD10: F10-F19) was defined as the date of the first diagnosis. Our classification of attempted suicide was identical to that used previously [19], using different algorithms for different time periods.

#### Criminality

The National Crime Register became electronic in November 1978, and all judicial verdicts and police decisions relating to criminal charges have been registered since [20]. It contains information on date of verdicts (or police decision), type of offence, verdict and sentence. Data have been made available to researchers through Statistics Denmark from 1980 onwards. We defined interpersonal violent criminal offending as encompassing all sexual offences (except for possessing child pornographic material), homicide, assault, robbery, aggravated burglary or arson, possessing a weapon in a public place, violent threats, extortion, human trafficking, abduction or kidnapping, rioting, and terrorism. We defined a custodial sentence as one imposing mandatory detainment of the convicted individuals, either in prison or in some other closed therapeutic and/or education institution, such as a reformatory, or a forensic psychiatric or drug detoxification unit. Driving under the influence of alcohol or drugs was defined as all driving offences due to alcohol, narcotics, or non-prescribed medicine. For the criminality outcomes we considered the first offence after 15^th^ birthday, the age of criminal responsibility and registration of criminal charges and convictions in Denmark.

### Study design and statistical analyses

Cohort members were followed up from their 15th birthday, and follow-up was terminated at the outcome of interest, death, emigration from Denmark, 40^th^ birthday, or December 31^st^ 2011, whichever came first. The cohort was followed for a total of 28.9 million person years. We estimated sex- and age-specific incidence rates and cumulative incidences for first occurrence of each outcome of interest. While the incidence rate measures the number of persons with the adverse outcome for the first time in their life per 10,000 person-years at risk, the cumulative incidence, also known as absolute risk, measures the percentage of persons in the population who will have the adverse outcome before a given age. Age-specific cumulative incidences were calculated for each sex and adverse outcome separately using competing risk survival analyses to account for the fact that persons are simultaneously at risk of experiencing an adverse psychiatric or criminality outcome, dying, or emigrating from Denmark. Ignoring censoring from emigration and/or death will bias the estimated incidence rates downwards and the estimated cumulative incidences [21]. The direction of bias for the latter depends on the nature of the censoring mechanisms.

## Results

**Fig. 1:**
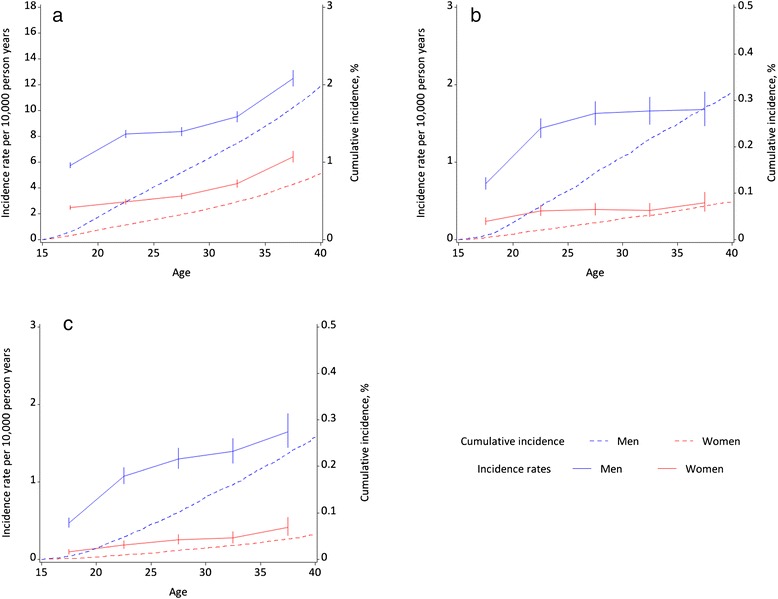
Sex- and age-specific incidence rates and cumulative incidence (absolute risk) for mortality outcomes: **a** All-cause mortality; **b** Suicide; **c** Accidental death

**Fig. 2:**
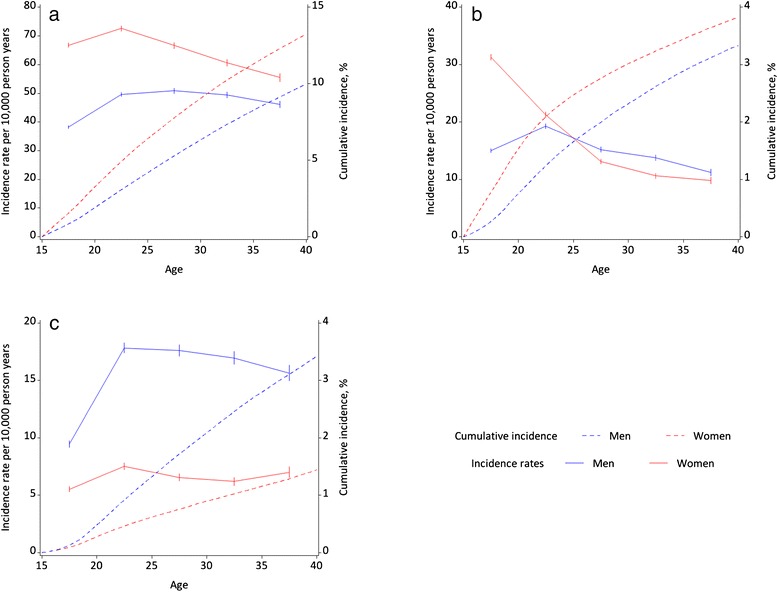
Sex- and age-specific incidence rates and cumulative incidence (absolute risk) for psychiatric morbidity outcomes: **a** Any psychiatric disorder; **b** Suicide attempt; **c** Alcohol or drug misuse disorders

**Fig. 3:**
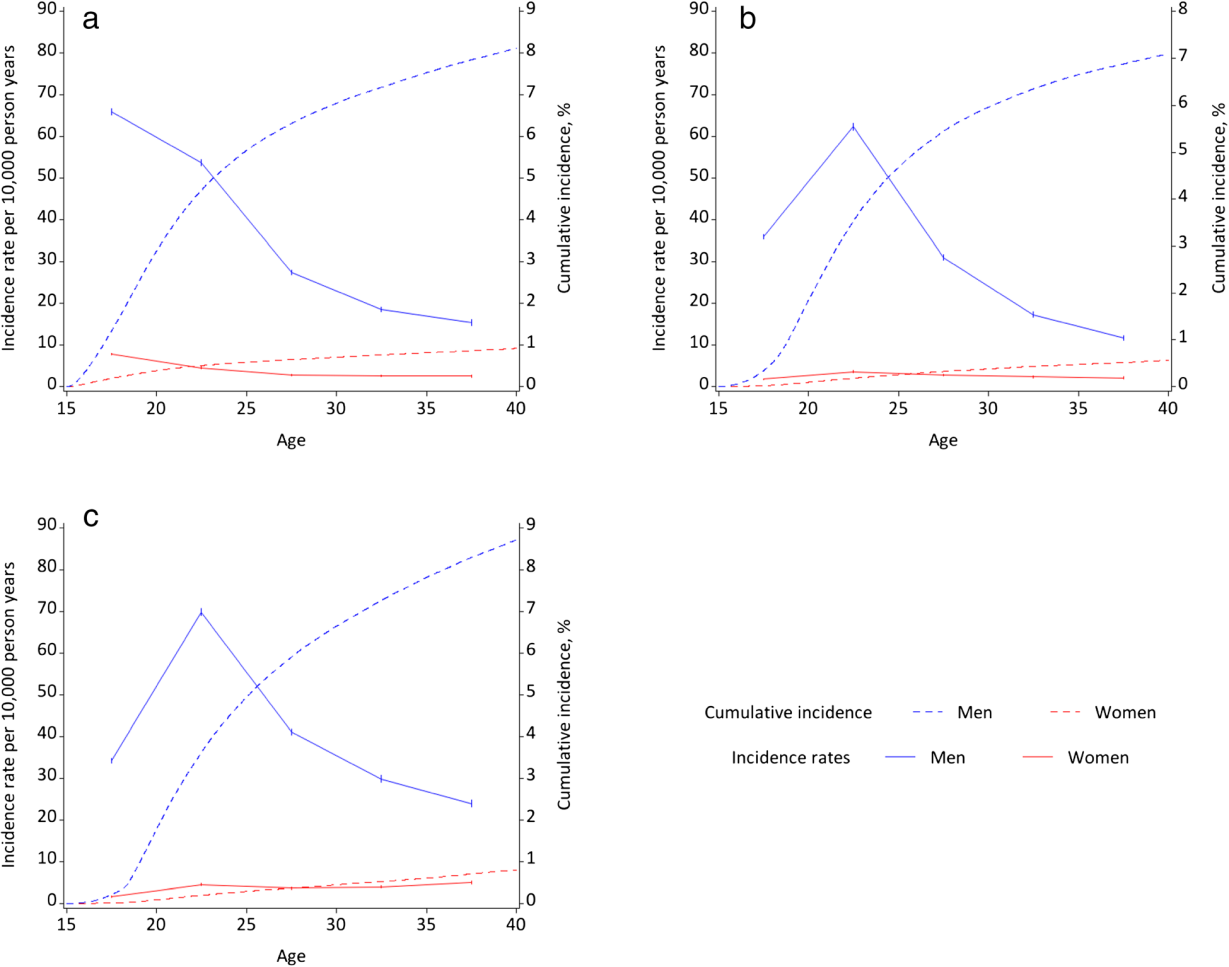
Sex- and age-specific incidence rates and cumulative incidence (absolute risk) for criminality outcomes: **a** Violent offending; **b** Custodial sentence; **c** Driving under the influence of alcohol or drugs

Figures 1, 2 and 3 show the sex- and age-specific incidence rates and cumulative incidences for the nine adverse outcomes under the three categories of *Premature mortality*, *Psychiatric morbidity*, and *Criminality*, respectively. The number of incident cases for each outcome by sex and the sex-specific cumulative incidence by the 40^th^ birthday are shown in Table 1. The differences in sex-specific incidence rates by age for each outcome are shown in Table 2.

**Table 1:** Number of incident cases of each adverse outcome and estimated cumulative incidences by 40^th^ birthday

	Number of cases of adverse outcome between ages 15 and 39 years	Cumulative incidence as a percentage (95 % CI) by the 40^th^ birthday
Premature mortality		
All causes		
Male	12004	1.99 (1.95–2.03)
Female	4709	0.85 (0.83–0.88)
Suicide		
Male	1941	0.32 (0.30–0.33)
Female	473	0.08 (0.07–0.09)
Accidental death		
Male	1525	0.26 (0.25–0.28)
Female	280	0.05 (0.05–0.06)
Psychiatric morbidity^a^		
Any psychiatric illness		
Male	65574	9.98 (9.91–10.06)
Female	87465	13.25 (13.16–13.33)
Attempted suicide		
Male	23001	3.33 (3.29–3.38)
Female	27948	3.83 (3.79–3.88)
Alcohol or drug misuse disorders		
Male	21830	3.42 (3.38–3.47)
Female	8910	1.44 (1.41–1.48)
Criminality		
Violent criminal offence		
Male	63778	8.12 (8.05–8.18)
Female	6685	0.92 (0.90–0.95)
Custodial sentence		
Male	53363	7.10 (7.04–7.16)
Female	3554	0.56 (0.54–0.58)
Driving under the influence of alcohol or drugs		
Male	61748	8.72 (8.65–8.79)
Female	4571	0.80 (0.77–0.82)

^a^Cases identified from secondary care registers only

**Table 2:** Differences in sex-specific incidence rates per 10000 person years by age between males and females^a^

	15–19 years	20–24 years	25–29 years	30–34 years	35–39 years
Premature mortality					
All-cause mortality	3.3	5.3	5.0	5.2	6.1
	(3.0 to 3.5)	(4.9 to 5.6)	(4.6 to 5.4)	(4.7 to 5.7)	(5.3 to 6.8)
Suicide	0.5	1.1	1.2	1.3	1.2
	(0.4 to 0.6)	(0.9 to 1.2)	(1.1 to 1.4)	(1.1 to 1.5)	(1.0 to 1.5)
Accidental death	0.4	0.9	1.0	1.1	1.2
	(0.3 to 0.4)	(0.8 to 1.0)	(0.9 to 1.2)	(0.9 to 1.3)	(1.0 to 1.5)
Psychiatric morbidity					
All psychiatric illnesses	−28.5	−23.0	−15.8	−11.2	−9.3
	(-29.5 to -27.6)	(-24.1 to -21.8)	(-17.1 to -14.5)	(-12.7 to -9.7)	(-11.1 to -7.5)
Attempted suicide	−16.3	−2.0	2.1	3.1	1.4
	(-16.9 to -15.7)	(-2.6 to -1.3)	(1.5 to 2.7)	(2.5 to 3.8)	(0.6 to 2.2)
Alcohol or drug misuse disorders	3.9	10.3	11.1	10.8	8.6
	(3.6 to 4.3)	(9.8 to 10.8)	(10.5 to 11.7)	(10.1 to 11.4)	(7.8 to 9.5)
Criminality					
Violent criminal offence	58.1	49.2	24.7	15.9	12.9
	(57.3 to 58.9)	(48.5 to 50.0)	(24.1 to 25.4)	(15.2 to 16.5)	(12.1 to 13.6)
Custodial sentence	34.2	58.8	28.2	15.0	9.6
	(33.6 to 34.7)	(58.0 to 59.7)	(27.5 to 28.9)	(14.4 to 15.6)	(8.9 to 10.2)
Driving under influence of alcohol or drugs	32.6	65.4	37.5	26.0	19.0
	(32.1 to 33.2)	(64.6 to 66.3)	(36.7 to 38.3)	(25.1 to 26.8)	(18.1 to 20.0)

^a^A positive value denotes that the incidence rate for male is greater than that for female, while a negative value denotes the opposite

For those who were alive on their 15^th^ birthday, the absolute risks of dying prematurely by age 40 were 1.99 % for males [95 % CI (confidence interval) 1.95–2.03 %] and 0.85 % for females (95 % CI 0.83–0.88 %). Deaths by suicide were more common than from accidents. For both sexes, incidence rates for suicides and accidental deaths doubled between ages 15–19 and 20–24 years, with the rates of increase falling thereafter. Deaths from all causes occurred most commonly at ages 35–39 years. For all three mortality outcomes, males had higher incidence rates than females across all age groups, with the gender differentials rising sharply between ages 15–19 and 20–24 years, and showing little change thereafter (Table 2).

Of the nine adverse outcomes examined, for both males and females, the highest absolute risks by 40^th^ birthday were for receiving secondary care level treatment for a mental disorder. Females showed higher incidence rates than males for this outcome across all age groups, although the differentials became smaller with increasing age. For both sexes, incidence rates for treated psychiatric disorder rose between 15–19 and 20–24 years of age. While incidence rates decreased for females after age 25 years, there was little change in the incidence rates for males.

Females also showed a higher absolute risk than males of attempting suicide by age 40 years. Women aged 15–19 years were the most likely to first attempt suicide compared with women in other age groups, while incidence rates for men peaked at age 20–24 years. At age 15–19 years, the incidence rate of suicide attempts for females was double that of males. Women aged 20–24 years also had higher incidence rates of attempted suicide than men of the same age, but the gender differential was much reduced from that at age 15–19 years. From age 25–29 years, the pattern was reversed with males showing higher incidence rates of attempted suicide than females. Men, however, were more likely than women to be treated for an alcohol or drug misuse disorder at all ages. In particular, there was a sharp rise from 9.5 to 17.8 per 10,000 person years in the incidence rates for young males between ages 15–19 and 20–24 years, while rates for females rose from 5.5 to 7.5 per 10,000 person years between the same ages. The increases in sex-specific incidence rates between these two youngest age groups were also accompanied by a widening of the gender differentials.

The risks of having a criminal conviction were also much higher for men than for women. Across all age groups, men and women aged 15–19 years were the most likely to be convicted of a first violent crime, with the incidence rates for males falling substantially between ages 15–19 and 25–29 years. The gender difference in age-specific incidence rates for violent offending was greatest at age 15–19 years, but fell steadily with increasing age. On the contrary, young males and females aged 20–24 years were the most likely to receive a custodial sentence or be convicted of driving under the influence of alcohol or drugs, compared with their counterparts in other age groups. The gender differentials in incidence rates for these two outcomes also rose sharply between ages 15–19 and 20–24 years, and then fell with increasing age. Table 2 shows that across all nine adverse outcomes examined, the greatest gender differentials in observed age-specific incidence rates were for the three criminality outcomes.

## Discussion

### Main findings

In a national cohort of adolescents, youths and younger age adults we estimated sex- and age-specific absolute risks and incidence rates for nine adverse outcomes related to premature mortality, psychiatric morbidity and criminality occurring between 15^th^ and 40^th^ birthdays. While males were more likely than females to die prematurely, and specifically, by suicide or accidents, women were more likely to have a secondary care treated psychiatric disorder across all age ranges examined. Women under 25 years of age were more likely than men to first attempt suicide, but these gender-specific patterns were reversed for cohort members older than 25 years. Males were also more likely than females to have treated substance misuse disorders and have a criminality outcome. Males aged below 20 were the most likely to be convicted of a first violent offence, and those aged 25–29 years the most likely to be given a custodial sentence or be convicted of driving under the influence of alcohol or drugs. Across all adverse events examined, the greatest gender differentials in incidence rates were for the criminality outcomes.

### Evidence from existing research and interpretation

This is the first study to report absolute risks and incidence rates for these multiple adverse events in the same complete population cohort at risk. Comparisons of our figures with other studies are difficult due to the differences in study designs, periods, age ranges, population covered, and measures reported [22–25]. However, it has been well-established in almost all countries, that while men are more likely than women to die by suicide, women attempt suicide more frequently [26, 27]. Our figures from a national cohort followed from mid-adolescence to early middle age additionally showed that while females had a much higher rate of suicide attempt than males during the teenage years, the sex-specific incidence rates actually converged with increasing age and finally crossed over by mid-late 20s. Researchers in the UK have previously reported that the rate ratios for female:male self-harm cases fell from 3.1:1 for 15–19 years to 1.3:1 for 25–49 years old. Only from age 50 years onwards was the rate ratio reversed with males having the higher gender-specific rate [28]. However, unlike our study, which was based on a national cohort and included incidence cases only, the previous study was based on data collected through one general hospital and included both incidence as well as repeated cases presented to this hospital in different years. Since having a previous episode of suicide attempt is one of the greatest risk factors for a subsequent attempt [29], if our study had included repeated cases, the gender and age profiles may be different from those reported here.

Adolescent males were more likely than any other groups we examined to be perpetrators of violent crimes. Previous studies have reported that both prevalence and incidence of offending peaked during adolescence (at around age 17 years) before falling in young adulthood [30, 31]. Our research has also shown that the gender differentials in being convicted of a serious crime varied with age. Furthermore, by examining the same young cohort at risk across multiple adverse outcomes, we have found that the gender differentials in criminality incidence rates were far greater than those for mortality and psychiatric morbidity adverse outcomes.

Females were more likely than males to have had a hospital-treated psychiatric disorder. Earlier research using the Danish registers has found that, over the life course, women were more likely than men to be diagnosed with anxiety, mood disorders, and eating disorders, while men were more likely to be diagnosed with autism, mental retardation, hyperkinetic disorders, and schizophrenia, in addition to substance use disorders [32]. We also found a large increase in the incidence rates of alcohol and drugs misuse disorders and conviction for driving under the influence of substance misuse between ages 15–19 and 20–24 years, and large differences in the gender patterns. The legal age of driving is 18 years in Denmark, which may partially explain the higher incidence rate for driving under the influence of alcohol or drugs at age 20–24 years versus 15–19 years. Within the World Health Organization European Region, heavy episodic drinking has been reported to be more prevalent among younger than older people, especially for males [33]. However, while birth cohort effects were not investigated in our study, others have reported increases in substance use among younger age women and a converging gap between male and female substance use and associated disorders [25, 34, 35]. We may therefore see an increase in incidences of substance use disorders and any other substance-related adverse outcomes amongst females in the future.

Our study did not investigate the mechanisms underlying the similarities and differences in the profiles of these multiple adverse outcomes. However, comparison of profiles of incidence rates suggests that adolescence and early adulthood are particularly vulnerable periods for many of these adverse events. Major brain and other biological and psychosocial developments occur during adolescence and puberty, with associated emotional and behavioural changes [36]. For males, the especially large increases in incidence rates for suicide and accidental death between ages 15–19 and 20–24 years coincided with the large rises in incidence rates for alcohol or drug misuse disorders and any psychiatric disorders, both of which have been identified as key factors associated with these two predominant causes of unnatural death [3, 37, 38]. It has also been reported that earlier and later onset of suicidal behaviours exhibit different aetiologies and risk profiles [39–41]. Using a life course approach, a study of suicide cases with a mean age of death at 37 years has identified two distinct suicide trajectories [41]. While there were common risk factors for the two groups in their early life histories (e.g. being a victim of physical and/or sexual abuse), the group who died by their early 20s showed higher levels of conduct, behavioural, and social difficulties during development. Conversely, the second group experienced lesser adversities and a slower decline before taking their own lives at a later age.

Psychopathology and substance misuse are also known risk factors for suicide attempts and violent offending [42–44]. Although our study has shown a higher incidence rate for suicide attempts for males at age 20–24 years compared with 15–19 years, risks for attempted suicide in females and for violent offending in both genders fell markedly between adolescence and early adulthood, which did not match the trends in incidence rates for any psychiatric or alcohol and drug misuse disorders. Pubertal changes, especially during the later stage of puberty, have been linked to increased risks for both suicidal and non-suicidal self-harm with the associations being much stronger for girls than for boys [45, 46]. With better emotional regulation and stress coping strategies associated with increasing age, most of these self-destructive behaviours resolve spontaneously by young adulthood [46]. Previous studies have also suggested different mechanisms underlying early- and late-onset of violent offending, and that much of the antisocial behaviours during adolescence is motivated by the gap between biological and social maturity [31, 47]. Further research is necessary to investigate the aetiology contributing to the similarities and differences in the onset profiles of these adverse outcomes and the differentials in gender patterns.

### Strengths and limitations

One of the main strengths of our study was its prospective design, a complete national cohort that provided abundant statistical power and precision for examining rare adverse events. Unlike sampled population surveys, we faced no issues of recall or selection bias, due to participants’ unwillingness to report suicide attempts, mental illnesses, or criminal offences, for example. The Danish CRS also contains complete information on migration, allowing such censoring events to be accounted for [21]. In addition, other published reports on incidence and risk often focus narrowly on a single outcome, making comparisons of these different endpoints difficult due to different settings and study designs. By examining the same cohort at risk, not only could we directly compare absolute risks and incidences for these multiple adverse outcomes across three different domains (premature death, psychiatric morbidity and criminality), we were also able to compare the age-specific incidence rates between males and females both within and across the array of outcomes.

Our study also had some limitations, however. Firstly, whilst there is complete nationwide registration of all patients treated in psychiatric units, milder cases of mental disorders, attempted suicides, and substance misuse treated in primary care settings may not be recorded in the Danish Psychiatric Central Research Register. Hence, our figures for the three psychiatric morbidity outcomes only included cases toward the more severe end of the disorder spectrum. There have also been changes in psychiatric practice over the decades, from inpatient care to a shift towards more outpatient and community-based care. Our follow-up period spanned from 1981 (when those born in 1966 became aged 15 years) to 2011, and outpatient data have only been available since 1995. Therefore a second limitation of our study could be potential cohort effects due to the heterogeneity of the psychiatric data availability and changes in psychiatric care practice. A third possible limitation is that our findings may not generalise to other countries, such as the United States where rates of violent criminality and custodial sentencing are far higher than in Denmark [48]. However, as some of the patterns in age- and sex-specific incidences that we found concur with those reported from previous studies [26, 27, 30, 31], it is likely that these patterns would also apply to other western European countries.

## Conclusions

This nationwide study provides a first comprehensive assessment of the incidence rates and absolute risks of multiple adverse outcomes. For the nine adverse events examined, males and females showed significantly different incidence rates and, in some cases, distinctive patterns by narrower age group. A sizeable minority of the population are at considerable risk of an adverse psychiatric or criminality outcome before reaching 40 years of age. These epidemiological profiles will guide aetiological research into the causes of and pathways leading to these harmful events, and the future development of integrated preventive strategies to tackle multiple adverse outcomes. They also will usefully inform clinicians and public health experts specialising in mental health, substance misuse and adolescent health and wellbeing, as well as professionals working in the social services, law enforcement and the criminal justice system.

## Abbrevations

CI: Confidence interval
CRS: Civil Registration System
ICD: International Classification of Diseases
ICD-10-DCR: International Classification of Diseases, 10th revision, Diagnostic Criteria for Research
